# Application of the Mirror Technique for Three-Dimensional Electron Microscopy of Neurochemically Identified GABA-ergic Dendrites

**DOI:** 10.3389/fnana.2021.652422

**Published:** 2021-04-20

**Authors:** Petra Talapka, Zsolt Kocsis, Lívia Diána Marsi, Vera Etelka Szarvas, Zoltán F. Kisvárday

**Affiliations:** ^1^MTA-DE Neuroscience Research Group, University of Debrecen, Debrecen, Hungary; ^2^Department of Anatomy, Histology and Embryology, Faculty of Medicine, University of Debrecen, Debrecen, Hungary

**Keywords:** mirror technique, electron microscopy, dendritic synaptome, 3D-TEM, GABA interneurons, calbindin

## Abstract

In the nervous system synaptic input arrives chiefly on dendrites and their type and distribution have been assumed pivotal in signal integration. We have developed an immunohistochemistry (IH)-correlated electron microscopy (EM) method – the “mirror” technique – by which synaptic input to entire dendrites of neurochemically identified interneurons (INs) can be mapped due preserving high-fidelity tissue ultrastructure. Hence, this approach allows quantitative assessment of morphometric parameters of synaptic inputs along the whole length of dendrites originating from the parent soma. The method exploits the fact that adjoining sections have truncated or cut cell bodies which appear on the common surfaces in a mirror fashion. In one of the sections the histochemical marker of the GABAergic subtype, calbindin was revealed in cell bodies whereas in the other section the remaining part of the very same cell bodies were subjected to serial section EM to trace and reconstruct the synaptology of entire dendrites. Here, we provide exemplary data on the synaptic coverage of two dendrites belonging to the same calbindin-D_28__K_ immunopositive IN and determine the spatial distribution of asymmetric and symmetric synapses, surface area and volume of the presynaptic boutons, morphometric parameters of synaptic vesicles, and area extent of the active zones.

## Introduction

Neurons typically receive synaptic input on their dendrites from presynaptic boutons, about hundred times more than on the cell body. Dendrites, even if they are passive, integrate input signals non-linearly, thus the relative position of synapses gives rise to a salient influence regarding input-output function (Koch et al., 1982). Therefore, a deep understanding of how input synapses are distributed on the dendritic tree is essential for predicting accurately the resulting responses at the cell body level. Despite the wealth of theoretical and modeling efforts which emphasize the computational power along the soma-dendrite domain the precise distribution of synapses and their interaction which result in changes in the membrane potential according to the input load is still elusive (Poirazi and Papoutsi, 2020). What is more, neuroanatomical data as to the compartmentalization of synaptic input onto interneurons (INs) is still in its infancy.

It has been conjectured that in the cerebral cortex each neuron type is implicated in specific circuit functions, i.e., the input components along the dendrites are characteristic for the particular neuron type (Karimi et al., 2020). However, this basic tenet has not been validated experimentally due largely to technical difficulties. In this regard the closest study so far aimed to disclose how synaptic input is distributed along the entire length of vasoactive intestinal polypeptide (VIP) positive INs. They used confocal microscopy (Sohn et al., 2016), which although returned positional information about the synapses along the dendrites, however, without morphometric parameters which in fact are accessible only by using electron microscopy (EM) (Domínguez-Álvaro et al., 2020; Montero-Crespo et al., 2020).

For revealing the chemical phenotype of a neuron, light microscopic detection of neurochemical markers is commonly used. However, investigating synaptic connections of the same, chemically characterized structures the tissue must meet very strict requirements. From a technical point of view only good ultrastructure can warrant continuous tracing and fault-free identification, especially of those small structures such as dendritic processes. Recent large-scale efforts have been challenging to map synaptic connections using three-dimensional (3D) volume reconstruction of neural tissue: transmission EM (TEM) tomography (Bates et al., 2020; Marin et al., 2020), focused-ion-beam Scanning EM (Bosch et al., 2015; Xu et al., 2017), and block-face-SEM (Kubota et al., 2018a; Wool et al., 2019). Commonly, high quality tissue fixation was employed which allowed tracing fine neuronal processes from section to section provided that the cell membranes were well preserved. Novel correlative light microscopy (LM)-EM methods can pave the way for ensuring this criterion and allow precise volumetric 3D reconstruction of dendrites and their synaptic elements (Thomas et al., 2020). Theoretically, the best tissue preservation is achieved when the fixation process is not compromised by other treatments, such as immunohistochemistry (IH) and fluorescent labeling. These techniques often require the use of chemicals such as detergents which reduce the integrity of membranes. As a generalization, any extraneous treatment of the tissue decreases fidelity of cell membranes. Hence, histochemical and immunostaining protocols bear conflicting requirements to combine with EM investigations (Micheva et al., 2010; Oberti et al., 2011; Collman et al., 2015).

**FIGURE 1:**
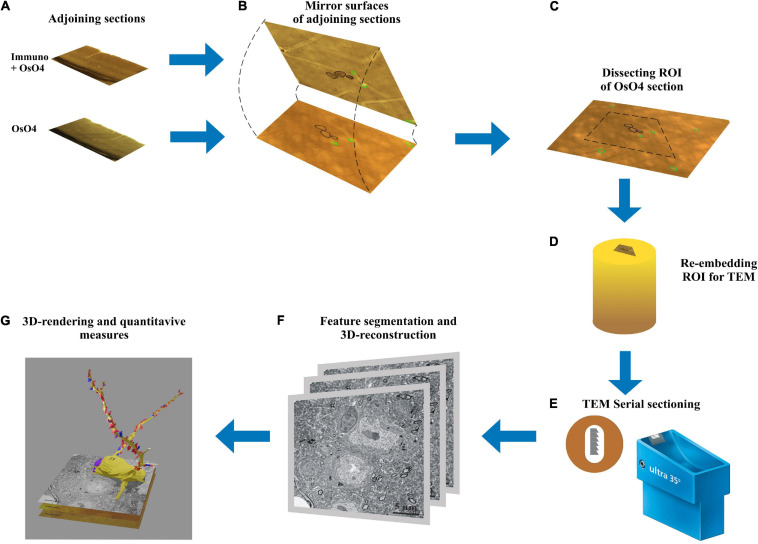
Workflow of the mirror-technique indicating major steps from identifying immunolabeled neuronal cell bodies (section: Immuno + OsO_4_) and determining their dendritic synaptome in the adjoining section (section: OsO_4_). **(A)** Adjoining sections underwent different procedures, one for revealing a neurochemical for light microscopy, and the other for examining electron microscopic attributes. **(B)** Neuronal cell bodies cut by the sectioning plane offer correlation between their neurochemically identified nature and synapse distribution of the dendritic processes. **(C)** Once immunopositive cell bodies have been selected on the section surface the corresponding region of interest (ROI) containing the same cell bodies is determined in the OsO_4_ section. **(D)** The ROI is trimmed and re-embedded in a block for EM sectioning. **(E)** Long series of sections are cut and picked up on Formwar-coated single slot grids. **(F)** Each section is inspected under TEM and dendrites originating from the selected cell bodies are traced and photographed for quantifying synaptic input distribution. **(G)** After alignment and segmentation of EM images a three-dimensional reconstruction of the dendrites is generated revealing from synaptic coverage of entire dendrites.

Here, we report an application of the mirror-technique (Kubota et al., 1985) which solves the above conflicts. For this purpose, detection of the IN marker and the subsequent EM analysis was carried out in two separate but adjoining sections. The mirror-technique is based on the following rational: cell bodies, which are cut on the surface can be subjected to immunohistochemical detection of neuronal markers while the mirror-section for EM analysis (see workflow in Figure 1). Important to note that the mirror-section is nascent because it is treated only with osmium prior to dehydration thereby its ultrastructure is not compromised. Consequently, 3D mapping of synapses within the mirror-section can be carried out for the entire length of dendrites from soma origin to natural termination. The mirror-technique not only provides quasi-optimal tissue condition to perform reliable and fault-free serial EM reconstruction of the dendritic synaptome, but also compatible with multiple histochemical or genetic (Livet et al., 2007; Sakaguchi et al., 2018) identification of neuron types in the other section. This technical approach offers superb quality for TEM/SEM investigations and through this can disclose hidden topographical aspects of synapses which can be exploited in biologically realistic models of dendritic signal integration (for review, see Poirazi and Papoutsi, 2020).

## Materials and Methods

Animals were maintained and bred in the animal house facility of Department of Anatomy, Histology and Embryology under appropriately controlled conditions with approval of Local Ethics Committee for Animal Research Studies at the University of Debrecen in line with European Union guidelines for the care of laboratory animals (Directive 2010/63/EU).

### Tissue Fixation

Anesthetized (Urethane, 1.5 g/kg) 14-weeks-old C57BL/6J male mice (*n* = 6) was transcardially perfused having clamped the descending aorta with Tyrode’s solution (gassed with 97% O2 and 3% CO2; pH = 7.2) for 1 min followed by fixative containing 2% paraformaldehyde, 1% glutaraldehyde and 15% (v/v) saturated picric acid in 0.1 M Phosphate Buffer (PB; pH 7.4) for 40 min (rate: 3 ml/min). Then the brain was removed and stored in the same fixative at 4°C overnight. It should be noted that the method introduced here was carried out successfully on all six animals however, to clarifying the technical details in coherent manner data are presented for the same animal.

### Sample Preparation for Pre-embedding Immunohistochemistry

**FIGURE 2:**
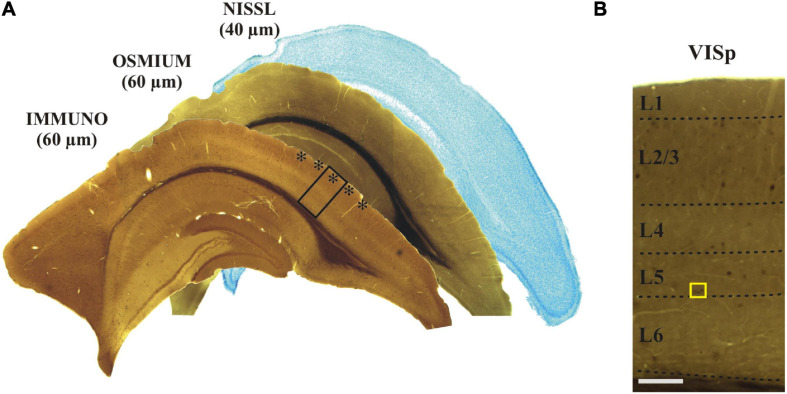
**(A)** Adjoining coronal sections were processed for immunostaining, treated with osmium and stained with Nissl, respectively. The primary visual cortex (VISp, asterisks) was determined according to the Allen Brain Atlas. The framed area is shown at higher magnification in panel **(B)** indicating (yellow rectangle) those layer five calbindin-D_28__K_ immuno-positive interneurons which were selected for EM analysis. Scale bars: 1 mm **(A)**; 100 μm **(B)**.

After rinsing the brain in 0.1 M PB (pH 7.4), tissue blocks containing the region of interest [ROI: primary visual cortex area (VISp), see Allen Mouse Brain Atlas; Lein et al., 2007] were dissected using a Mouse Brain Matrice (Electron Microscopy Sciences, Mouse Coronal, 69090). From each block 60 μm thick coronal sections were cut on a vibratome (LEICA, VT 1000S) and collected in sequential order in 0.1 M PB (pH 7.4) at room temperature (RT; 22-23°C). Altogether 20–25 sections could be made from the tissue block of which alternate sections were subjected for IH and every other section was processed for EM (without IH). For controlling rostro-caudal position and identifying the cortical area (Allen Mouse Brain Atlas), every sixth section was stained with Cresyl-violet (Figure 2A).

### Pre-embedding Immunostaining

For demonstrating the viability of the mirror-technique, Calbindin-D_28__K_ antibody was chosen as a reliable and widely used marker of cortical INs (del Río and DeFelipe, 1997). All steps were carried out at RT unless otherwise stated. In order to reduce endogenous peroxidase activity sections were treated with 1% H_2_O_2_ diluted in 0.05 M Tris-buffered-saline (TBS; pH 7.6) for 15 min. Next, a mixture of 0.25% bovine-serum-albumin, 0.1 M DL-Lysin and 10% normal goat serum (Sigma-Aldrich, G9023) in 0.05 M TBS (pH = 7.6) containing 0.05% Triton X-100 was used (1 h) for suppressing non-specific immunoreactivity. Thereafter, the neuron-type marker, monoclonal antibody to calbindin-D_28__K_ (Swant, 300; supplemented with 0.05% Triton-X 100) was applied in 1:2,000 dilution for 2 days at 8°C during continuous agitation of the sections. Secondary antiserum, biotinylated goat anti-mouse IgG (VECTOR Laboratories, BA-1000) was used in 1:200 dilution for 4 h, followed by avidin-biotin complexed to horseradish peroxidase (ABC; VECTOR Laboratories, PK-6100) in 1:400 dilution for overnight. Between each step sections were washed in 0.05 M TBS (pH 7.6) 3 × 10 min. For visualization of the immuno-labeling, sections were incubated in 0.05 M 3,3-diaminobenzidine-tetrahydrochloride (DAB; Sigma-Aldrich, D-5637) in 0.05 M Tris Buffer (TB; pH = 7.6) for 10 min. Completion of the enzymatic reaction was made in the presence of 0.02% H_2_O_2_ for 1–2 min followed by 2 × 5 min rinse in TB.

### Post-fixation

Post-fixation protocol was applied for all sections including immuno-labeled (L) and alternate non-immuno-labeled (NL) sections. All steps were carried out at RT. Sections were washed in TB (3 × 10 min) followed by modified Millonig’s PB (2 × 15 min, MPB: containing 1.66% monobasic sodium phosphate and 2.52% sodium hydroxide in 100 mL with added 5% glucose) and post-fixed in osmium-tetroxide (1% OsO_4_ diluted in MPB). For L sections osmium was used for 10 min and for NL sections for 30 min. After a thorough wash in MPB (3 × 15 min), sections were dehydrated in increasing concentration of ethanol (50, 70, 96, and 100%, 2 × 10 min each) followed by propylene oxide (2 × 10 min) and flat embedded on slides in resin (DurcupanTM ACM; Sigma-Aldrich, 44610).

### Identification of Immuno-Labeled Neurons Using the “Mirror” Technique

**FIGURE 3:**
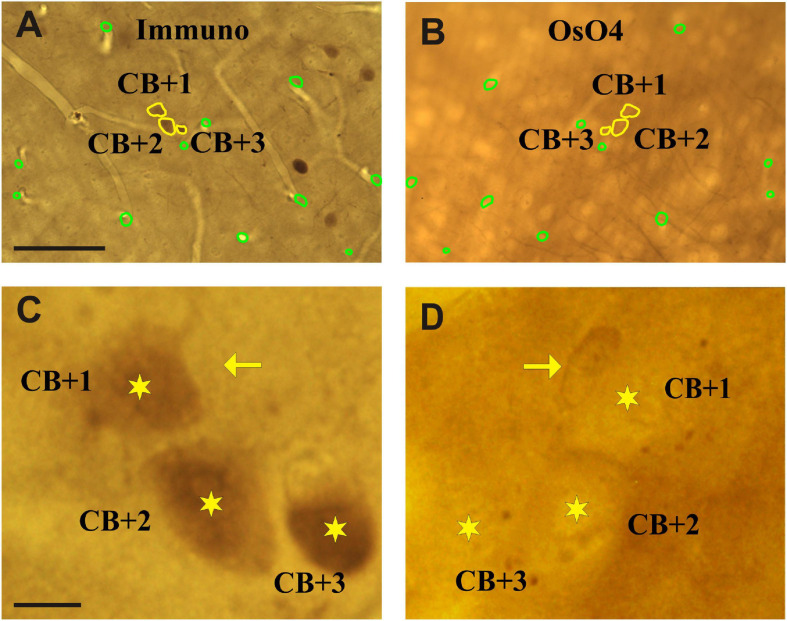
Light micrographs of adjoining sections viewed from their facing surfaces which represent mirror images. **(A)** Calbindin-D_28__K_ immuno-labeled somata are indicated by the peroxidase reaction end-product (brown). The contour (yellow) of three immunolabeled somata which were cut by the vibratome’s blade is marked (CB + 1–3). **(B)** Complementary parts of the same somata are seen on the mirror surface of the non-immunolabeled adjoining section. Fiducial landmarks such as cut edges of blood vessels are indicated by green contours. **(C,D)** Enlarged views of the above three cell bodies (stars) as seen on the mirror surfaces. The silhouette of a nearby astrocyte is indicated by an arrow. Scale bars: 50 μm **(A,B)**; 5 μm **(C,D).**

Neuronal cell bodies which are cut by the vibratome blade can be identified on the surface of adjoining sections (mirror sections). In our case one part of the cell body on the surface of the L section and the remaining part on the surface of the section prepared for EM. The contour of immuno-positive somata and various fiducial landmarks (e.g., truncated blood vessels) were drawn on the section surface using x100 oil objective and the Neurolucida (v.8.5) neuron reconstruction system (Figures 3A,B). Next, the above contours were overlaid with the mirror surface of the adjoining section and the layout of the very same cell bodies were determined using the silhouette of somata (Figures 3C,D). In addition to the computer aided cell body and landmark contours, it is recommended to do a thorough photo-documentation of the two mirror surfaces for clarity. Then a ROI (about 1 × 1 mm^2^) was defined in the NL section and this region, which contained the pre-selected INs, was cut out using a sharp-pointed scalpel. In order to avoid breaking the section during carving the scalpel in the rigid tissue the section had been warmed up to 40–42°C on a hot plate. The section part, typically less than a 1 mm^2^ in size, was transferred on a glass slide and an EM block prepared according to the re-embedding protocol described by Somogyi and Freund (1989).

### Preparation of the EM Block

**FIGURE 4:**
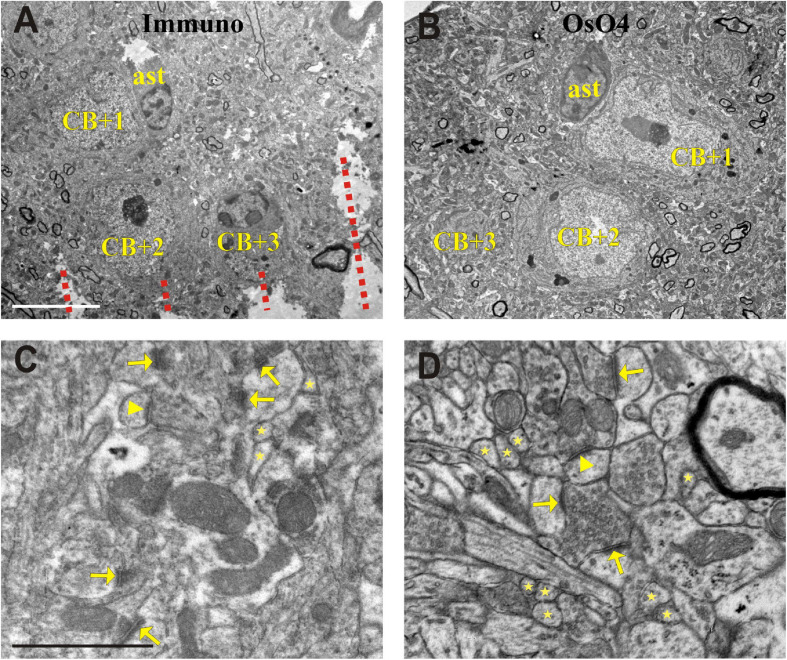
Electron micrographs showing somata (CB + 1–3) in the section processed for immunohistochemistry (Immuno + OsO_4_) **(A)** and their complementary parts in the mirror section treated only with OsO_4_ **(B)**. Representative images of the neuropil in the Immuno + OsO_4_ **(C)** and OsO_4_ alone **(D)** sections to demonstrate ultrastructural superiority of the latter. The cell membranes are less intact in panel **(C)** and hence inappropriate for fault-free tracing of small and fast-changing profiles (stars) and unequivocal identification of asymmetric (arrows) and symmetric (arrowheads) synapses. The integrity of membranes in the tissue without immunohistochemistry **(D)** allows 3D reconstruction of even the smallest structures and a clear identification of symmetric (arrow-head) and asymmetric (arrows) synapses which is rather compromised in panel **(C)**. Broken lines in panel **(A)** indicate Vibratome chatter whose presence signified section surface. ast = astrocyte; Scale bars: 5 μm **(A,B)**; 1 μm **(C,D)**.

Identifying the selected INs at the surface of the block before ultrathin sectioning is the first and mandatory step because nearby neuronal cell bodies can be confounded. Then, the wall of the EM block is trimmed to finalize its shape commonly a trapezoid along the sectioning plane. Using the above-mentioned high-power photographs as a reference, the layout of IN cell bodies and nearby blood vessels are determined on the block surface as indicated by the Vibratome chatter pattern (Figure 4A). For further control, it is recommended to take LM images of the block surface, too. After identification of the selected INs and photo-documentation were completed, ROI defined in the NL section was cut out and embedded to EM block as well.

### Preparation of Ultrathin Sections for Three-Dimensional Reconstruction

The truncated cell body of the selected CB + INs was located on the surface of the EM block. Since one of the main aims was to reconstruct only those dendrites, which are emitted from the designated INs cell body it was utmost importance to start collecting EM sections from the very surface of the block using an Ultracut 6 (Leica) microtome. First, the overlying few μm resin that covered the block surface was carefully removed by taking 0.5 μm sections and staining with Nissl on slide until the cell body of the IN was reached. Once the IN soma was reached sectioning continued and serial sections of 50 nm were cut. A total of 1,200 ultrathin sections comprising the entire thickness of the vibratome section (60 μm) were collected on Formwar-coated (Polyvinyl formal; Polysciences, 00631) single-slot nickel grids (6–10 sections/grid) and stained with Reynolds’ lead citrate solution for 10 min (Reynolds, 1963). Care was taken to keep the sequential order of the sections. To this end a single series containing no more than 30 sections in a ribbon was gently divided with an eye-lash in shorter segments. i.e., 6–10 sections, and picked up on grids, respectively. For registry purposes, the layout of sections on each grid was photographed using a Leica M80 stereo microscope fitted with an OCS-SK2-10X digital microscope camera (Optixcan). Every 20th or so ultrathin section was placed on a grid alone for determining GABA-content of the structures (see below).

### Post-embedding GABA Immunostaining

Here, we used the protocol introduced by Somogyi and Hodgson (1985). Briefly, all steps were carried out at RT on droplets of reagents placed on a parafilm sheet (Parafilm M Film; Amcor, PM992) in a petri-dish. Sections were pre-treated with 1% periodic acid for 9 min, washed 3 × 5 min in distilled water (DW) followed by 2% sodium-periodate for 10 min. Next, they were washed 3 × 5 min in DW and 1 × 5 min in 0.05 M TBS (pH 7.4). For reducing non-specific binding of the primary serum 1% ovalbumin in 0.05 TBS (pH 7.4) was used for 30 min followed by washing in 0.05 M TBS (pH 7.4) for 2 × 10 min. Primary antiserum against GABA (Sigma-Aldrich, A2052) was diluted in 0.05 M TBS (pH 7.4) at 1:1,000 and applied for 90 min. Sections were washed for 2 × 10 min in 0.05 M TBS (pH 7.4) and then in 0.05 M TBS (pH 7.47) containing 2% BSA and 0.5% Tween 20 for additional 10 min. The secondary antiserum, 15 nm colloidal gold particles conjugated to goat anti-rabbit IgG (BBI Solutions, EM.GAR15) was diluted in the latter buffer at 1:40. After 90 min incubation in the secondary serum, sections were washed 3 × 5 min in DW. Contrasting the sections was made by floating the grids on uranyl acetate (2.5% in 25% ethanol) droplets for 20 min, washed 1 × 5 min in 50% ethanol and 3 × 5 min in DW and exposing to Reynolds’ lead citrate solution for 10 min. After a thorough wash in DW for 3 × 5 min the sections were air-dried.

**FIGURE 5:**
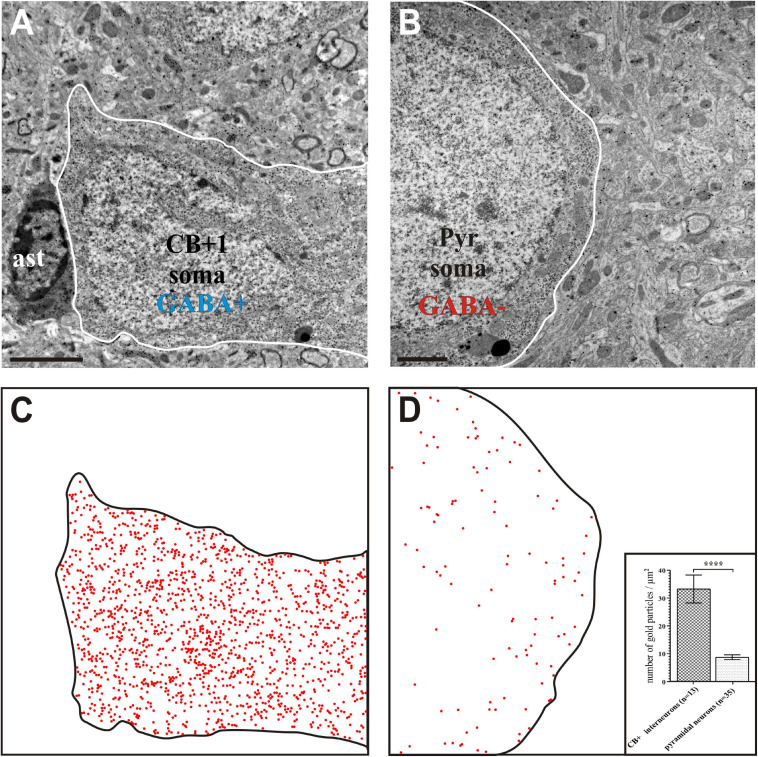
Gamma-aminobutyric acid (GABA) immunohistochemistry using the post-embedding colloidal gold method. Interneuron CB + 1 **(A)** contained high density of gold particles indicating positive GABA-immunostaining whereas the soma of a pyramidal (Pyr) neuron **(B)** contained gold particles only at the background level indicating GABA immuno-negativity. **(C,D)** Schematic figures in order to visualize the presence of nano-gold particles above CB + 1 and Pyr cell bodies (contour lines). Each red dot represents an immuno-gold particle. Note the high density of gold particles in the CB + 1 soma **(C)** whereas their density reflects background level in the Pyr soma **(D)**. Inset in panel **(D)** shows statistical comparison between the GABA labeling intensity of cell bodies identified as CB+ and pyramidal shaped, respectively (*n* indicates the number of TEM micrographs analyzed; data are expressed as mean ± SEM; ^****^ *p* < 0.0001). Scale bars: 1 μm **(A,C)**; 2 μm **(B,D)**.

Post-embedding GABA immunostaining was used for validation the inhibitory characteristic of CB + INs, the density of gold particles in somata of CB + INs and presumed pyramids was determined on three ultrathin sections (Figure 5). In every section, counting of gold particles was performed on non-overlapping TEM micrographs taken from the CB + 1, 2, and 3 as well as five neighboring pyramidal neurons in Image J (1.46r). Utilizing GABA gold labeling on approximately every 20th ultrathin section was proven to be helpful in confirming classification as putative excitatory or inhibitory presynaptic boutons.

### Electron Microscopy and Image Processing

**FIGURE 6:**
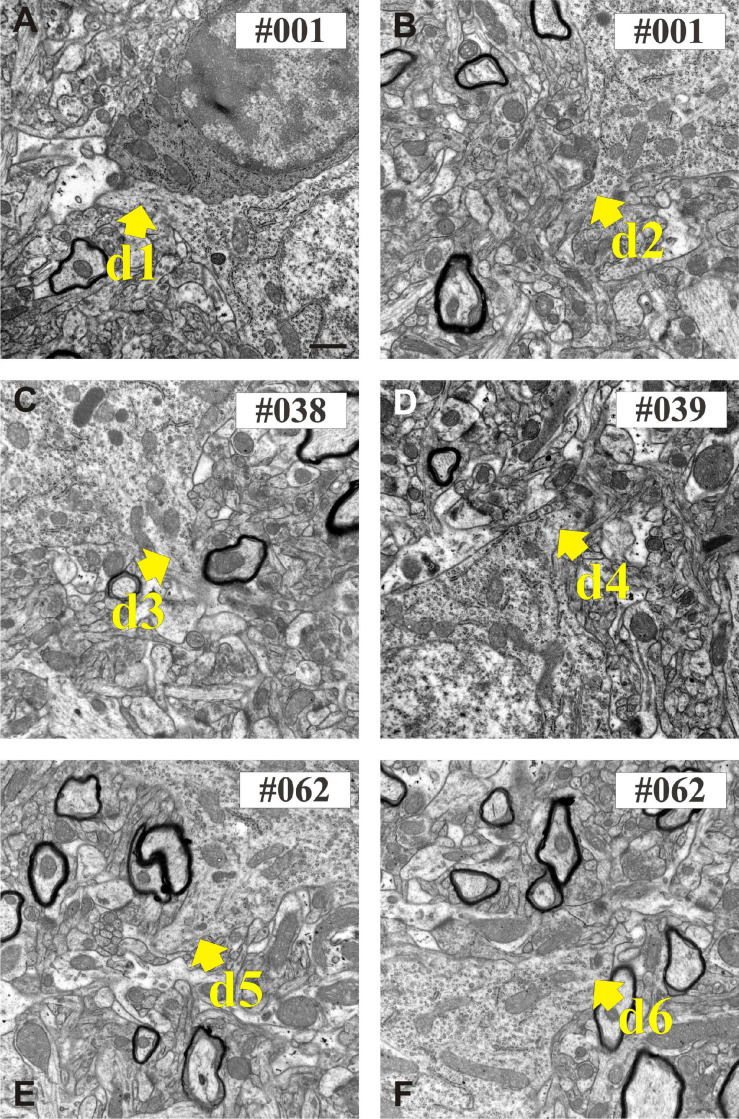
Electron micrographs show the proximal part of dendritic shafts (den1–den6) emerging from the calbindin-D_28K_ (CB + 1) immuno-positive cell body **(A–F)**. Numbers indicate sequential position in the section series. Scale bars: 500 nm.

Ultrathin sections were examined and photographed using a JEOL-1010 TEM equipped with a digital camera (Olympus Veleta). Sections were examined in a sequential order although occasionally re-examination of the sections was necessary. The dendritic profiles of the INs were photographed at two magnifications in each section; a low resolution image (×10,000, 7.13 nm/px) for documenting the neighboring neuropil and a high resolution image (×30,000, 2.39 nm/px) for documenting structural details of presynaptic boutons in association with the traced dendrites (Figure 6).

### Three-Dimensional Volume Reconstruction

**FIGURE 7:**
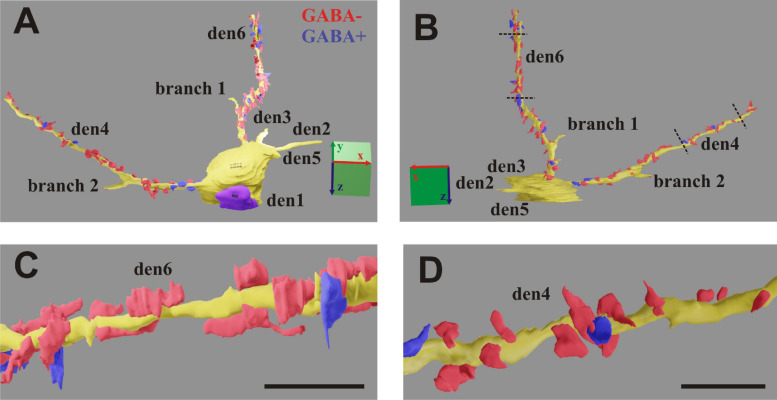
**(A,B)** 3D-rendering of the EM reconstruction of a calbindin-D_28K_ immuno-positive interneuron (CB + 1). Two dendrites (den4 and den6) were reconstructed up to the distal end, while another four dendrites (den1, den2, den3, and den5) and two side branches only partially. For den4 and den6, presumed inhibitory boutons establishing symmetric type of synapse (GABA+) are marked in blue, while presumed excitatory boutons establishing asymmetric type of synapse (GABA−) are marked in red. **(C,D)** Zoom-on reconstruction of den6 and den4 as delimited by broken lines in panel **(B)**. Scale: 5 μm **(A,B)**; 1 μm **(C,D)**.

TrakEM2 (Cardona et al., 2012) software (graphical interface of Image J 1.46r) was used for creating image stacks in two steps. First, all EM images were calibrated and spatially aligned (pairwise matching) using only linear transformation (rotation and shift) of image overlays. Second, feature segmentation such as contour of designated dendrites, presynaptic boutons and synaptic active zones were carried out for the entire image stack (Figure 7; Supplementary Movies 1, 2). The extent of synaptic zones (synaptic cleft) in association with presynaptic boutons was determined based on the presence of post-synaptic density (PSD) and the accumulation of synaptic vesicles (Peters et al., 1991). Characterization of synapse type such as type I or asymmetric (*as*) and type II or symmetric (*ss*) was made according to morphometric parameters and with the help of GABA labeling when available. Synapses which could not be classified unequivocally (intermedier type) were assigned to the type I category. The contour of presynaptic boutons either “en passant” or “terminaux” was drawn within 1 μm radius centered to the active zone. Main dendrites were traced and segmented from soma origin through the 60 μm thick tissue blocks. Daughter branches, having smaller diameters than the main dendrite, were traced only for a limited distance from the branch points, i.e., until they became separated from the main dendrite by twice the image field size. Since the EM protocol described here allows re-examination of the EM sections tracing of dendritic branches can be completed any time upon need.

### Compression Correction of Ultrathin Sections

**FIGURE 8:**
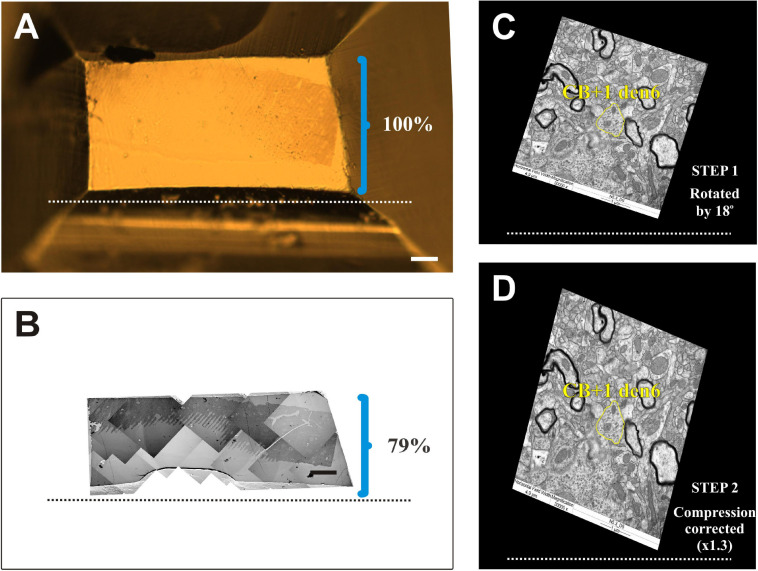
Compression correction of ultrathin sections. **(A)** Surface view of an EM block (resin-embedded “mirror” section). **(B)** Photo montage of electron micrographs using ultrathin sections from the surface of the block. The knife edge is indicated by broken lines. Brackets in panels **(A)** and **(B)** mark the hight of block surface and that of the sections, respectively, perpendicular to knife edge. Clearly EM sections suffer from considerable (21%) compression. **(C,D)** One dimensional compression of EM sections needs to be corrected in order to retain due spatial dimensions of the imaged structures. Accordingly, EM images are rotated (STEP 1) in line with the knife edge as exemplified in panel **(C)** and then expanded (STEP 2) perpendicular to the knife edge (broken line) by an empirically determined compression factor. Note the difference in the shape of the dendritic profile (yellow contours) belonging to the calbindin-D_28__K_ immuno-positive interneuron dendrite (CB + 1 den6) before and after correction. Scale bars: 50 μm **(A,B)**.

It has been known that resin embedded tissue sectioned for EM suffers from compression exerted by the knife edge (Jésior, 1986). In order to determine the compression factor, the aspect ratio of block surface area was compared with that of 50 nm thick sections taken at regular intervals (every 50th section) of the same block. On average, 21% compression was observed in the direction perpendicular to the cutting edge while parallel to it no change at all was seen (Figures 8A,B). Accordingly, compression correction was performed *post hoc* by rotating the entire EM reconstruction of IN dendrites together with accompanying synaptic structures in the plane parallel to the sectioning plane and multiplying the coordinates values by a factor of 1.3 perpendicular to the axis of the knife edge (Figures 8C,D).

### Morphometric Analysis

**FIGURE 9:**
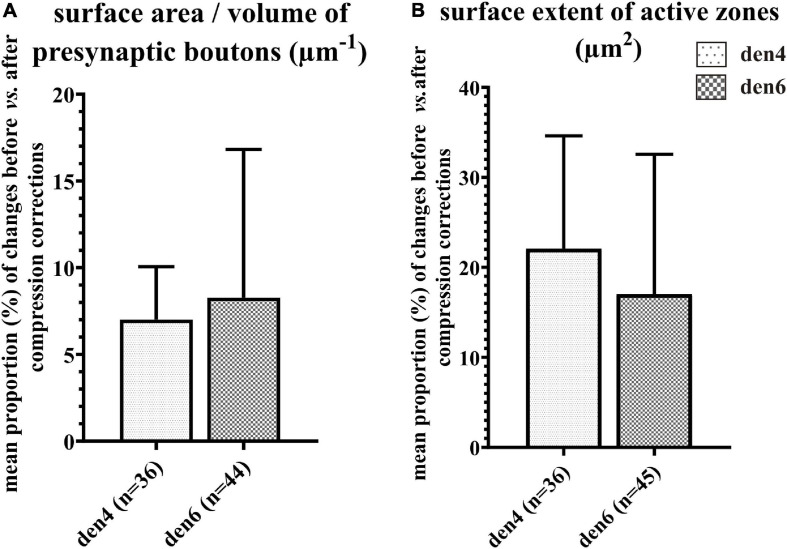
Impact of compression correction on the morphometric parameters of presynaptic boutons and synaptic active zones. Changes in morphometric parameters before and after compression corrections are expressed as percentage for individual presynaptic boutons and their active zones. Mean proportional values (%) of surface area/volume ratio of presynaptic boutons **(A)** and area extent of synaptic active zones **(B)** are compared between den4 and den6 (*n* indicates number of boutons and active zones; data area expressed as mean + SD).

**FIGURE 10:**
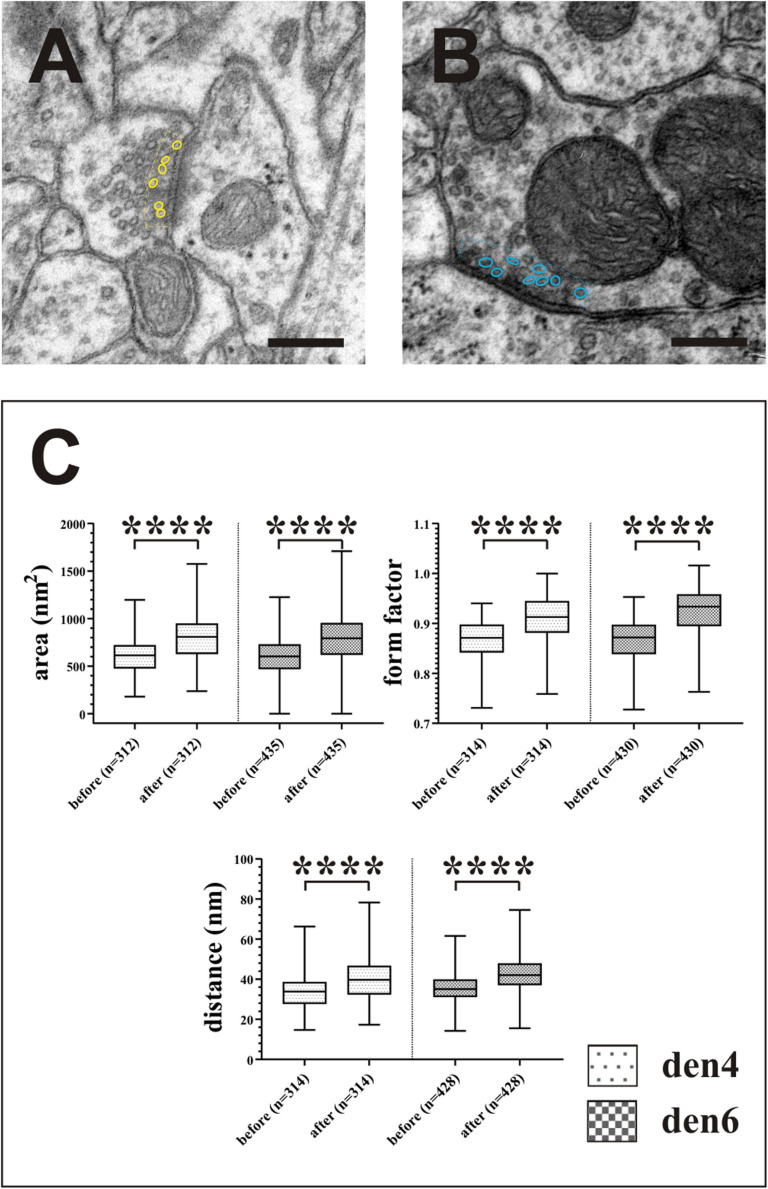
Impact of compression correction on the morphometric parameters of synaptic vesicles. Vesicles located no farther than 50 nm (broken lines) from the active zones of asymmetric [**(A)**, yellow outlines] and symmetric [**(B)**, blue outlines] synapses were included in the statistical analysis **(C)**. Compression gives rise to significant alterations in quantitative measures of area, form factor and spatial separation of synaptic vesicles. ^****^p < 0.0001; Scale bars: 200 nm.

Right after 3D-reconstruction, volume and surface area of dendrites and presynaptic boutons, and surface area of the active zones of synapses were exported before and after performing compression corrections. Presynaptic boutons whose reconstruction could not be completed mainly at truncated parts of the dendrites were not included in statistical analyses (den4: 5 as; den6: 2 as, 3 ss) (Figure 9). For determining the morphometric parameters of synaptic vesicles, single EM micrographs taken at high magnification (80,000×) were chosen for each presynaptic bouton. Since synaptic vesicles representing those of immediate use are typically attached to the plasma membrane by ∼20–60 nm filaments (Figures 10A,B; Burette et al., 2012), the contour of vesicles located less than 50 nm from the active zones were drawn using Neurolucida (MBF) reconstruction system. Measurements were performed according to the Marker and Region analyses tool by which area (nm^2^), form factor and centroid (*x*, *y*, *z*) values of contours were determined. From the *X*, *Y* centroid values of vesicle contours the nearest neighbor distance (nm) of vesicles was derived.

### Statistical Analyses

For statistical analyses, GraphPad Prism (v.8.0.0.) software was used. In every case, Shapiro-Wilk normality test was used to determine whether the datasets fit Gaussian distribution.

Density of gold particles reflecting GABA immunopositivity of CB + INs was demonstrated on bar chart after Mann–Whitney *U* test was performed and data are expressed ± SEM (Figure 5D insert).

Difference in morphometric parameters of boutons and synaptic active zones gathered before vs. after performing compression corrections are demonstrated on bar charts with standard deviations as means of percentage values obtained from each bouton (Figure 9). Parameters of synaptic vesicles were demonstrated on boxplots (Figure 10C) after removing outliers (*Q* = 1%; ROUT method). Choosing probes for pairwise comparison of related quantitative morphometric data collected before vs. after compression corrections depended on the distribution characteristic of the datasets (two-tailed paired *t*-test or Wilcoxon signed-rank test).

Significance for all tests was set at the threshold of *p* < 0.05.

## Results

For demonstrating the potential of the mirror-technique, 6 dendrites of three calbindin immunopositive cells (CB+) were studied in VISp (Figure 2). Serial section EM was carried out by tracing the dendrites from their soma origin. Figures 3A–D shows the cluster of the three CB + cell bodies at low (Figures 3A,B) and high magnification (Figures 3C,D) right on the surface of adjoining sections representing the immunolabeled (L) and the non-immunolabeled (NL) or mirror-section, respectively. Preservation of the ultrastructure of tissue block deriving from L section was unsuitable for fault-free tracing of small and fast-changing dendrite profiles as compared with that of NL section (Figure 4). Obviously, in the L section much of the membranes lost structural integrity which prevented falt-free tracing of neural processes across hundreds of section, in particular for those small diameter dendrites. It should be pointed out that synapses represented the most resistant locations where membrane integrity suffered the least possibly due to the high density of proteins associated with the pre- and post-synaptic zones holding their together. An EM investigation of the three CB + cell bodies displayed that their ultrastructural features are characteristic for GABAergic neurons such as high density of endoplasmic reticulum, numerous mitochondria, the presence of nuclear folds and relatively few axo-somatic synaptic contacts. The GABA content of all three CB + and 4 additional immuno-labeled neurons was verified using post-embedding GABA-immunolabeling in the cell bodies (Figure 5) and dendritic processes. On the contrary, none of the nearby 6 pyramidal neurons with a distinct apical dendrite showed positive immunostaining to GABA (Figure 5, see inset to Figure 5D).

### Serial Section Electron Microscopy of CB + Dendrites

Six dendrites (den1-den6) of CB + 1 neuron were chosen for serial sectioning in order to determine their presynaptic bouton distribution from soma origin. Figure 6 shows exemplary EM images of the 6 dendrites at the position where they emerged from the parent cell body. The dendrites were photographed in all sections at medium (×4,000 and ×10,000) and high magnification (×30,000) in order to obtain an overview of ROIs as well as structural details of the input synapses. For the latter, typical image size was 2,048 × 2,048 pixels using 2.39 nm per pixel resolution (×30,000). In this way, identification of the type of synaptic contacts could be readily made and intracellular organelles within the presynaptic boutons and post-synaptic dendrites determined.

Section alignment and image segmentation of pre- and post-synaptic structures (see section “Materials and Methods”) was carried for all six dendrites and presynaptic boutons. A total of 1,174 sections were photographed and assembled in a single image stack corresponding to 58.70 μm, i.e., the entire thickness of the vibratome section. The ultrastructural quality of the tissue allowed obtaining quantitative measurements, which are known to be important in modeling dendritic signal integration mechanisms.

### Quantitative Morphometry

The number of synaptic contacts (symmetric, ss, GABA+ vs. asymmetric, as, GABA- or inhibitory vs. excitatory) were estimated based on the 3D reconstructions (Table 1).

**TABLE 1:** Number of excitatory and inhibitory boutons encountered for two dendrites (den4 and den6) of calbindin-D_28K_ immuno-positive interneuron CB + 1.

Branch number	GABA+		GABA−	
	Number	Ratio (%)	Number	Ratio (%)
den4	35	85	6	15
den6	37	76	12	24

In order to estimate the impact of the compression correction of ultrathin sections, morphometric parameters gathered before performing compression corrections were compared to those after corrections (see Table 2): differences were determined as mean percentage values regarding surface/volume ratio of the presynaptic boutons (μm^–1^) [den4: 7 % (SD: 3.06) vs. den6: 8.27 (SD: 8.55)] (Figure 9A) and surface extent of active zones (μm^2^) [den4: 22.08% (SD: 12.54) vs. den6: 17.06% (SD: 15.54)] (Figure 9B); mean area of synaptic vesicles [den4: 616.7 nm^2^ (SD: 191.1) before vs. 811.7 nm^2^ (SD: 251.5) after; den6: 612.3 nm^2^ (SD: 186.4) before vs. 802.9 nm^2^ (SD: 241.2) after] (Figure 10C), form factor of synaptic vesicles [den4: 0.8657 (SD: 0.0404) before vs. 0.904 (SD: 0.0464) after; den6: 0.8649 (SD: 0.0429) before vs. 0.9229 (SD: 0.0495) after] (Figure 10C), nearest neighbor distance of synaptic vesicles [den4: 33.7 nm (SD: 8.298) before vs. 39.79 nm (SD: 9.841) after; den6: 36 nm (SD: 8.047) before vs. 43.23 nm (SD: 9.933) after] (Figure 10C). Significant differences were observed in every case (^****^p < 0.0001). As a result of compression correction all of the morphometric synaptic parameters increased significantly except the surface/volume ratio of presynaptic boutons which in turn decreased significantly.

**TABLE 2:** Effect of tissue compression on basic spatial parameters of 3D-reconstructed dendrites (den4 and den6) of calbindin-D_28K_ immuno-positive interneuron CB + 1.

	Dendrite with branches			
	Before correction		After correction	
	Length (μm)	Surface/volume ratio	Length (μm)	Surface/volume ratio
den4	40.62	1.2334	54.26	1.2273
den6	46.65	1.2838	66.25	1.2026

Moreover, average cumulative values of morphometric parameters gathered after performing compression corrections have been determined for all 6 dendrites to guarantee accurate classification: (1) surface/volume ratio of the presynaptic boutons (as: 11.4281 μm^–1^ vs. ss: 10.4812 μm^–1^); (2) surface extent of area of active zones (as: 0.0892 μm^2^ vs. ss: 0.0509 μm^2^); (3) area of synaptic vesicles (as: 832.1549 nm^2^ vs. ss: 698.4264 nm^2^); (4) form factor of synaptic vesicles (as: 0.9131 vs. ss: 0.9146) and (5) nearest neighbor distance of synaptic vesicles (as: 42.5717 nm vs. ss: 40.5341 nm) (data not shown).

Obviously, other morphological parameters, for example the spatial distribution profile of synapses along the dendritic tree, thickness and branching pattern of dendrites can be derived from the very same EM reconstruction which have been assumed essential in determining dendritic signal integration (e.g., Eyal et al., 2018; Iascone et al., 2020).

## Discussion

Mapping the synaptic organization of single neurons is an important step for understanding the principles of connectivity and circuit function. In this regard, EM provides the gold standard for the identification of synaptic contacts. Recently, there is an increasing demand in using EM techniques to shed light on the synaptology of entire structures rather than random sampling of neuronal structures. Technical difficulties arise when neuronal structures need to be viewed not only in isolation but in the context of all input synapses, for example, on entire dendrites. Specifically, the mirror-technique implemented for EM can solve correlate immunohistochemically identified IN types using 3D-EM of input synapses (synaptome) for entire dendritic processes. The rationale behind the mirror-technique is of two folds. First, high quality tissue ultrastructure is essential for TEM investigations to avoid the deleterious effect of immunohistochemical treatments. Second, an excellent ultrastructure is mandatory to trace dendrites across their entire length. It is known that dendrites of INs often show tortuous trajectories and may possess narrow segments occasionally less than 0.5 μm in diameter (Kubota et al., 2011). Therefore, IN dendrites can be reliably traced and reconstructed from serial sections only if the cell membrane is continuously intact and discernible, i.e., the tissue is well fixed for TEM. It is just conceivable that such a stringent ultrastructural criterion is necessary for tracing small structures that would not be reached using immunostaining protocols, irrespective how valuable information by EM-IH can offer, for example, receptor localization (Luján and Shigemoto, 2006; Booker et al., 2013).

The mirror-technique is based on the identification (match) of neuronal cell bodies which are cut across by the sectioning plane and thus their two halves are present on corresponding surfaces of the adjoining LM sections. In one of the LM sections immunohistochemical labeling of neuronal cell bodies is carried out while the adjoining section subjected to post-fixation solely for TEM analysis. Importantly, a precise match between the ROIs (or corresponding cell body halves) had to be achieved between the L and NL sections. To this the section surfaces were viewed using conventional transmitted LM. Needless to mention section thickness, especially following osmium treatment may compromise visibility and through this identification of the selected neuronal cell bodies. For 60–80 μm thick sections, 1% OsO_4_ still allowed trans-illumination so that the matching process could be performed.

The EM analysis was carried out for those dendrites, which emerged from the cell body-half located in the NL section. Since the same dendrites belong to a neuron for which the presence of the neurochemical marker, calbindin, has been verified the synaptome determined for the dendrites can be associated with the neurochemical nature of the parent cell.

### Tissue Shrinkage Due to Histological Protocols

Tissue shrinkage is determined by numerous factors such as fixation parameters and tissue embedding protocol. Tissue fixation protocol was identical for all animals and considered nearly isotropic (Fox et al., 1985). However, for the mirror-technique, adjoining sections underwent different histological protocols (immunostaining and post-fixation, respectively), consequently, shrinkage was determined for each section in order to match corresponding LM section surfaces with micrometer precision (see paragraph 5 of section “Materials and Methods”). For the mirror technique, adjoining sections underwent different histological protocols, consequently, shrinkage had to be determined for each section in order to match corresponding surfaces with nm precision.

### Shrinkage of EM Sections Due to Compression

During sectioning of resin embedded tissue for EM the specimen is exposed to a strong force acting perpendicular to the knife edge. Consequently, EM sections suffer from one dimensional compression whereby the height of EM sections is smaller than that of the tissue block. Although there are ways to attenuate the above compression effect, for example, briefly exposing the sections to xylene vapor before picking up on grid, shrinkage can still remain considerable, 20% or more. Such a 1D distortion needs to be considered and corrected for a faithful 3D reconstruction of all structures (see Table 2).

### Sectioning Plane of ROI

Light microscopic sections are commonly prepared in a plane (e.g., coronal) perpendicular to the cortical surface. This is largely so because most dendrites and axons entering/leaving the cortex are oriented radially, i.e., perpendicular onto the cortical surface. Indeed, for morphological classification, cortical neurons have been traditionally viewed in planes perpendicular to the cortical surface. On the other hand, the anatomical plane of EM samples is usually not taken into consideration. Instead, the plane of EM sections is important from the point of view of recognizing quasi-planar structures such the synaptic active zone which is best identifiable when the sectioning plane runs perpendicular to the synaptic cleft. Based on the spatial arrangement of input synapses along dendrites it is just conceivable the sectioning plane should be ideally perpendicular to the axis of the post-synaptic dendrite. Otherwise, the synaptic cleft will be viewed in an oblique plane or tangentially making synapse identification ambiguous and in turn can lead to difficulties in mapping the complete synaptic coverage. Apparently, dendrites can change their course, hence a general solution to the problem is not feasible. Nevertheless, taking into account the above considerations, dendrites of cortical neurons which typically run perpendicular to the cortical surface, a sectioning plane parallel to the cortical surface would be the favorable plane. It is thus recommended that future studies shall consider the orientation of dendrites by preparing samples, i.e., LM sections or EM tissue blocks, for mapping synaptic input of dendrites. Not only the orientation of dendrites in the specimen can influence the outcome of synaptic mapping but the particular dendrites under scrutiny. Recent investigations indicated that the basal dendrites of parvalbumin INs have distinct synaptic properties from those of apical dendrites (i.e., Cornford et al., 2019). It follows from this that the synaptome of a dendrite, be it completely determined, should be treated with caution and may not be generalized for other dendrites of the same IN. Needless to say, that the above considerations may be modified for other brain structures. Another aspect to discuss concerns the small diameter of dendrites that imposes another type of difficulty in 3D-tracing. Notably, when the diameter of dendrites commensurates with the thickness of the EM sections synapses may even go unnoticed due to the curvature of the active zone or the classification criteria as type I or II cannot be unequivocally applied. Last but not least, section thickness signifies the limit in sampling the full length of dendrites. From this it follows that the mirror-technique must take into account those spatial constraints imposed by the trajectory of dendrites in the specimen. Accordingly, a 60 μm thick section tends to unravel dendritic input constellation chiefly proximal to soma location. We estimate that for dendrites running nearly parallel to the plane of the section several hundred micrometer dendrite can be tackled some representing even the full length (see den4 in Figure 7).

### Time-Factor

An important aspect of any technical approach is time-consuming. In this respect the mirror-technique *per se* requires only a few hours of work to carry out full documentation and cell-to-cell correlation between adjoining surfaces. Specifically, within the ROI the contour of immunohistochemically labeled neurons and neighboring fiducial surface landmarks (e.g., capillary and section contours) are drawn at ×100 magnification using the Neurolucida reconstruction program which then overlaid with surface image of the adjoining section. For this purpose, graphics programs, for example, CorelDraw (Corel corporation, Canada) can be used. All other steps including data acquisition and subsequent morphometric analysis (image segmentation and quantification) typically take considerable time from months to years depending primarily on data volume.

### Alternatives, Pros, and Contras

Recent EM approaches using volume reconstruction offer alternatives through which histochemically identified neurons can be subjected for high through-put mapping of the dendritic input synapses. Serial section SEM technologies such as block face imaging can screen large enough areas for simultaneous mapping of several dendrites (mutli-ROI) and generate image stacks for which all levels are inherently aligned whereas using TEM technology each section must be aligned with its neighboring ones.

Obviously, there is a trade-off between the imaging area, resolution, and section thickness. Based on literature data, of the currently available SEM technologies FIB-SEM offers the highest voxel resolution in *z*-direction (15 nm, Knott et al., 2008), however, typically with a limited area size in the *xy* plane. Serial section block-face imaging can handle much larger ROIs in the *x–y* plane, although in *z*-direction the practical value is 40–50 nm. It should be noted that a clear advantage of SEM technologies compared with conventional TEM is that the former deals with tissue blocks which are free of spatial distortions, while the latter suffers from the occurrence of section artifacts such as wrinkles, folds and contamination by the contrasting medium. On the other hand, TEM offers re-examination of the specimen, therefore, structures which need repeated inspection, for example tracing dendritic branches, remain available. It should be mentioned that one of the special applications of ssSEM whereby sections are collected on a carbon tape and collected on wafers represents a compromise in offering large ROIs, relatively little section distortion, and repeated use of the specimen (Horstmann et al., 2012; Kubota et al., 2018b). Last but not least, at present, only those technologies which are based on the examination of sections instead of the block surface permit post-embedding EM-immunohistochemical detection of markers. In line with this, in the present study, sections at regular intervals could be tested for the presence of GABA, whereby putative inhibitory axon terminals establishing type II synapses along IN dendrites could be determined (see Figure 5). Although this has not been feasible yet using either the FIB-SEM or BF-SEM methods recent attempts showed promising results in combining pre-embedding immunolabeling with the latter SEM technologies (Rodriguez-Moreno et al., 2018; Boey et al., 2019).

## Conclusion

The major benefit of the mirror-technique is that the trade-off between neurochemical identification and tissue ultrastructure can be solved. The spatial separation of cell body characterization – using IH, transcriptomic analysis, *in situ* hybridization, fluorescent immunolabeling either alone or in combination – from 3D-TEM reconstruction of the dendrites increases the versatility of the technique by opening up new vistas in revealing synaptomes of IN subtypes. The great variability of the inhibitory neuron population detected, for example, by proteomic approaches put cell classification in a difficult position. Our method intends to exploit differential input organization between major inhibitory cell classes with the hope to gain insight regarding structural underpinnings of signal integration therein.

## Data Availability Statement

The raw data supporting the conclusions of this article will be made available by the authors, without undue reservation.

## Ethics Statement

The animal study was reviewed and approved by the Local Ethics Committee for Animal Research Studies at the University of Debrecen (2010/63/EU).

## Author Contributions

PT was responsible for coordination of the experiments, performed histology, data collection, and wrote the manuscript. ZsK participated in quantitative morphometry. LM performed the majority of 3D volume reconstructions. VS performed the histology and made ultrathin section series. ZK contributed to designing the project and participated in writing the manuscript. All authors contributed to the article and approved the submitted version.

## Conflict of Interest

The authors declare that the research was conducted in the absence of any commercial or financial relationships that could be construed as a potential conflict of interest.

## Supplementary Material

Supplementary Movie 1 Image stack covering CB + 1 den4 after segmentation.

Supplementary Movie 2 Three-dimensional reconstruction of CB + 1 interneuron with den4 and den6.
